# Association of temporal MASLD with type 2 diabetes, cardiovascular disease and mortality

**DOI:** 10.1186/s12933-025-02824-3

**Published:** 2025-07-15

**Authors:** Eugene Han, Kyung-Do Han, Yong-ho Lee, Kyung-Soo Kim, Sangmo Hong, Jung Hwan Park, Cheol-Young Park, on Behalf of Fatty LiverResearch Group of the Korean Diabetes Association

**Affiliations:** 1 Division of Endocrinology and Metabolism, Department of Internal Medicine, Keimyung University School of Medicine, Daegu, Korea; 2 Department of Statistics and Actuarial Science, Soongsil University, Seoul, Korea; 3 Division of Endocrinology and Metabolism, Department of Internal Medicine, Yonsei University College of Medicine, Seoul, Korea; 4 Division of Endocrinology and Metabolism, Department of Internal Medicine, CHA Bundang Medical Center, CHA University School of Medicine, Seongnam, Korea; 5 Division of Endocrinology and Metabolism, Department of Internal Medicine, Hanyang Guri Hospital, Hanyang University College of Medicine, Guri, Korea; 6 Division of Endocrinology and Metabolism, Department of Internal Medicine, Hanyang University College of Medicine, Seoul, Korea; 7 Division of Endocrinology and Metabolism, Department of Internal Medicine, Kangbuk Samsung Hospital, Sungkyunkwan University School of Medicine, 29 Saemunan-ro, Jongno-gu, Seoul, 03181 Korea

**Keywords:** Metabolic dysfunction associated steatotic liver disease, Type 2 diabetes, Cardiovascular disease, Mortality

## Abstract

**Background:**

We investigated the risk of type 2 diabetes (T2DM) and related comorbidities including cardiovascular disease (CVD), and mortality, based on changes in metabolic dysfunction associated steatotic liver disease (MASLD).

**Methods:**

We analyzed data from the Korean National Health Insurance Service for individuals aged ≥ 20 years. MASLD was defined as a fatty liver index (FLI), a prediction formula based on metabolic parameters, with a cutoff of ≥ 60. FLI measurements were compared within each individual over a 2 years period. Based on changes in FLI between two health checkups, individuals were classified into four categories; never MASLD (FLI consistently < 60), incident MASLD (FLI < 60 to ≥ 60), regressed MASLD (≥ 60 to < 60), and persistent MASLD (FLI consistently ≥ 60). The primary outcome was T2DM occurrence in the general population and myocardial infarction (MI), ischemic stroke, heart failure (HF) and mortality events in individuals with preexisting T2DM with adjustment for age, sex, smoking, alcohol drinking, and regular exercise.

**Results:**

In 4,397,808 individuals without T2DM, 229,475 (5.2%) developed T2DM during a median follow-up period of 7.3 years. The risk of incident T2DM was the highest in individuals with persistent MASLD compared to those who never had MASLD (HR = 5.28, 95% CI = 5.22–5.34). Individuals with incident or regressed MASLD also had increased risk of developing T2DM (HR = 3.30, 95% CI = 3.25–3.35 for incident MASLD, HR = 2.87, 95% CI = 2.82–2.92 for regressed MASLD). In a cohort of 636,520 individuals with preexisting T2DM followed for a median of 6.2 years, those with persistent MASLD had a higher risk of HF (HR = 1.28, 95% CI = 1.25 to 1.32), MI (HR = 1.15, 95% CI = 1.10 to 1.20), stroke (HR = 1.14, 95% CI = 1.09 to 1.19) and all-cause mortality (HR = 1.11, 95% CI = 1.09–1.14) compared to individuals who never had MASLD. Similarly, both incident and regressed MASLD were associated with an increased risk for HF, MI, stroke and all-cause mortality.

**Conclusions:**

Persistent MASLD is associated with an increased risk of incident T2DM, and further elevates the risk of CVD, and mortality among individuals with T2DM. Even individuals with incident or regressed MASLD exhibit an increased risk of these adverse outcomes compared to those who never had MASLD.

**Trial registration:**

N/A.

**Supplementary Information:**

The online version contains supplementary material available at 10.1186/s12933-025-02824-3.

## Background

Metabolic dysfunction associated steatotic liver disease (MASLD) is the most prevalent chronic liver disease, accounting for 32% worldwide [1, 2]. With increasing obesity, the incidence and prevalence of MASLD has increased substantially in the general population over recent years [3, 4]. Although the pathophysiology of MASLD is complex and heterogeneous [5], all phenotypes are associated with progressive liver injury, that can lead to advanced liver fibrosis, and hepatocellular carcinoma (HCC), and can also contribute to the development of other metabolic disorders, including cardiovascular diseases (CVD) [6–8]. In the US, MASLD is a principal cause of liver transplantation, and the number of liver transplantation recipients due to MASLD has been increasing [9].

Among metabolic diseases associated with MASLD, type 2 diabetes mellitus (T2DM) is one of the core metabolic dysfunctions, similar to obesity. The liver and pancreas play central roles in glucose and lipid metabolism; therefore, MASLD and type 2 diabetes share a complex and bidirectional relationship, with accumulating evidence suggesting that each condition may influence the development and progression of the other [5, 10–13]. Of note, in recent nomenclature, MASLD which was previous called nonalcoholic fatty liver disease more empathizes the role of glucose dysfunction than previous fatty liver disease definitions, involving prediabetes as an independent inclusion criterion [14]. When MASLD accompanies T2DM, it increases overall mortality and this association was also observed in individuals with prediabetes [15]. Moreover, T2DM is an important predictor for hepatic fibrosis in MASLD even in lean populations [16].

Although a close association between MASLD and T2DM has been reported, the preceding relationship between these diseases is not clear due to their complexity. Unlike T2DM, the status of MASLD can be verified. The changes in MASLD status can have various consequences. However, the data for hard outcomes including CVD and mortality associated with the changes in MASLD status is scarce. Thus, we aimed to investigate the risk of T2DM in the MASLD population by the changing status of MASLD using data from the Korean National Health Insurance Service (NHIS). In addition, we explored how changes in MASLD are associated with the risk of CVD, heart failure (HF), and all-cause mortality in the T2DM population.

## Methods

### Study populations

The current nationwide cohort study assessed data collected from participants in the National Health Insurance database maintained by the Korean NHIS. The Korean NHIS is a single insurer in the Korean public health insurance sector, which provides national health examinations for the Korean population [17, 18]. Nearly the entire Korean population attends health checkups conducted by the Korean NHIS; thus, the NHIS database can be used as a population-based national source to investigate various diseases [19]. The public health checkup is performed biannually and covers anthropometric measurements and laboratory tests after overnight fasting. Laboratory samples are collected and measured as described previously [3, 20]. This study complied with the Declaration of Helsinki; the study protocol was approved by the institutional review board (Soongsil University, SSU-202007-HR-236-01). Written informed consent was waived, as this study was based on deidentified administrative data. The dataset used in this study was obtained through a formal request to the Korean NHIS and was available to the investigators from September 7, 2020, to September 6, 2024. Due to the expiration of data access, no additional analyses could be performed beyond this period, and no intermediate datasets were retained by NHIS data policies.

### Study design

**Fig. 1:**
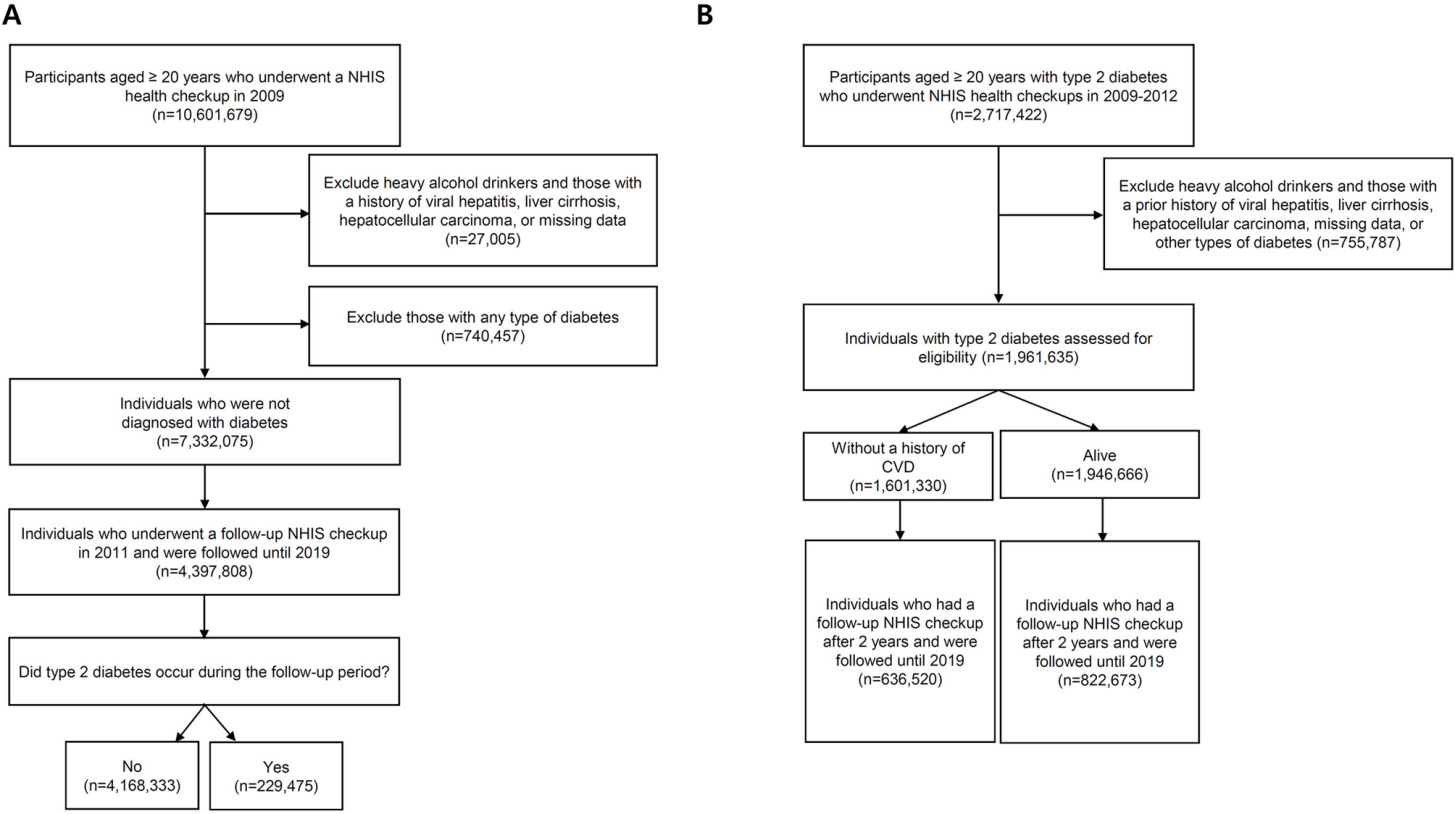
Study flow. **A** Study flowchart illustrating the incidence of T2DM in individuals with MASLD, and **B** study flowchart depicting the incidence of CVD and overall mortality in individuals with T2DM. *NHIS* national health insurance service,* T2DM* type 2 diabetes mellitus,* CVD* cardiovascular disease

The current study is composed of two parts. The first one is a longitudinal study to evaluate the incidence of T2DM by changes in MASLD status. We selected participants aged 20 years or older who had undergone a health examination in both 2009 and 2011, and they were followed until December 31, 2019. Participants with any type of diabetes and those with missing data were excluded. The exclusion criteria were as follows: (1) viral hepatitis (B15–B19), (2) liver cirrhosis (K703, K746), (3) HCC (C22.0), and (4) individuals with heavy alcohol consumption (≥ 30 g per day) (Fig. 1A). The lag period was set for 1 year to allow for a T2DM diagnosis.

The second part is a longitudinal analysis to determine the risk of myocardial infarction (MI), stroke, HF, and overall mortality in individuals with T2DM. To obtain information on incident comorbidities, we followed a cohort of 1,961,635 participants with T2DM who were aged 20 years or older and free from heavy alcohol drink, viral hepatitis, liver cirrhosis, or HCC (Fig. 1B). The index period was from January 1, 2009, to December 31, 2011. To assess the incident CVD and HF, individuals with previous CVD (composite of MI and stroke) or HF history were washed out. Follow-up health checkups were performed 2 years after the index date. Participants were followed until CVD, HF, the occurrence of death, or December 31, 2019 (Supplementary Fig. 1). We set the lag period for 1 year after each health checkup to allow for a disease diagnosis or death certificate.

### MASLD status definitions

The presence of steatotic liver disease is identified using fatty liver index (FLI), a well-validated and widely accepted noninvasive diagnostic tool [21]. FLI was calculated using the following formula: [e^0.953 × loge^ ^(triglyceride)+ 0.139 × body^ ^mass^ ^index + 0.718× loge^^(gamma glutamyltransferase)^ ^+ 0.053 × waist^ ^circumference −15.745^]/[1+e^0.953 × loge (triglyceride)^ ^+ 0.139 × body^ ^mass^ ^index + 0.718 × loge^ ^(gamma glutamyltransferase) + 0.053^ ^× waist^ ^circumference −15.745^] × 100. MASLD is defined based on the most recent guideline [14, 22]. The cut-off point is 60 for FLI, which was originally designed to include fatty liver disease [21], and was used to validate fatty liver disease in this analysis [23]. We defined individuals as four categories according to changes in the FLI between the 2 health checkups; never MASLD (FLI consistently < 60), incident MASLD (FLI < 60 to ≥ 60), regressed MASLD (FLI ≥ 60 to < 60), and persistent MASLD (FLI consistently ≥  60).

### Outcomes

T2DM was defined as one of the following: (1) at least one claim for a prescription of anti-hyperglycemic medication with the presence of International Statistical Classification of Disease and Related Health Problems, 10th Revision (ICD-10) codes E11–E14 either in outpatient or inpatient care, and (2) serum fasting plasma glucose level ≥ 126 mg/dL [24].

Ischemic stroke was identified based on inpatient records with an ICD-10 code of I63 or I64 in conjunction with claims brain imaging producers, such as computed tomography or magnetic resonance imaging [25]. MI was defined by the presence of ICD-10 code I12 or I22. HF was identified using the ICD-10 code I50.

Death certification was based on death data in the National Death Registry from the Korea National Statistical Office which is merged to the NHIS data of each individual. Individuals were followed until death or December 31, 2019. The validation of ICD-10 code in Korean NHIS were previously established [18, 26].

### Key variables

Hypertension was determined as ICD-10 codes I10 to I13, I15 plus treatment with antihypertensive agents, or systolic/diastolic blood pressure ≥ 140/90 mm Hg; dyslipidemia was ICD-10 code E78 plus treatment with lipid-lowering agents or total cholesterol ≥ 240 mg/dL. We considered those who receive medical aid or whose income was the lowest quartile in the study population as having low social economic status. Regular exercise was categorized as ≥ 5 times per week of moderate physical activity or ≥ 3 times per week of vigorous physical activity based on answers to questionnaires. Obesity was defined as ≥ 25 kg/m^2^ in body mass index based on the Asian-pacific cutoff values [27], and central obesity was defined as ≥ 90 cm and ≥ 85 cm in waist circumference for men and women, respectively [28]. Light alcohol drinkers were defined as individuals reported alcohol consumption of less than 30 g/day [14].

### Statistical analysis

Participant characteristics were presented as median (interquartile range), mean ± standard deviation, or number (%), as appropriate. Incidence rates were represented as events per 1000 person-years. Cox proportional hazards regression analysis was applied to identify the association between MASLD, T2DM, and cardiovascular comorbidities with adjustment for other covariates including age, sex, smoking, alcohol drinking, and regular exercise. CVD was assessed as a composite outcome encompassing ischemic stroke, MI and HF events. The results were presented as hazard ratios (HRs) and 95% confidence intervals (95% CI). Subgroup analysis by age groups (aged 20–39, 40–59, and > 60 years) and sex was performed to limit those factors in the association between MASLD, T2DM, and comorbidities. We reported effect estimates with 95% confidence intervals and interpreted* P* values as continuous indicators of statistical evidence, rather than relying on a fixed threshold for significance. Statistical analyses were performed using SAS version 9.4 (SAS Institute, Cary, NC).

## Results

### Baseline characteristics of the study population by incident T2DM

A total of 4,397,808 individuals (2,272,833 men and 1,895,500 women) without preexisting T2DM were included in the final analysis (Fig. 1A). During a median follow-up period of 7.3 years, 229,475 individuals (5.2%) were newly diagnosed with T2DM. The baseline clinical characteristics of the study population are summarized in Supplementary Table 1. At baseline, individuals who developed T2DM had higher fasting glucose levels and elevated liver enzymes, including aspartate aminotransferase, alanine transaminase, and gamma-glutamyl transferase, whereas their estimated glomerular filtration rate was lower (all *P* < 0.001). Those who developed T2DM were more likely to engage in regular physical activity but were less likely to be light alcohol drinkers.

### Risk of incident T2DM based on changes in MASLD status

Among the 229,475 participants who developed T2DM, 153,823 (67.0%) had never had MASLD, 18,662 (8.1%) developed incident MASLD, 15,920 (6.9%) experienced regressed MASLD, and 41,070 (18.0%) had persistent MASLD (Table 1). In the multivariate-adjusted model, the risk of incident T2DM increased progressively from regressed MASLD (HR = 2.87, 95% CI = 2.82–2.92) to incident MASLD (HR = 3.30, 95% CI = 3.25–3.35), with the highest risk observed in those with persistent MASLD (HR = 5.28, 95% CI = 5.22–5.34). Subgroup analysis demonstrated a similar trend in both men and women. When stratified by age, individuals with regressed or incident MASLD exhibited an elevated risk of T2DM, while the highest risk was consistently observed in those with persistent MASLD across all age groups. The risk of T2DM in persistent MASLD group was the most pronounced in younger individuals (20–39 years) with HR of 12.60 (95% CI = 12.25–12.96).

**Table 1:** Risk of incident T2DM by MASLD status change

	Groups	No. of incident T2DM/No. in group	Duration, years	IR (per 1000 PYS)	HR*	95% CI
Overall	Never MASLD	153,823 / 3,848,039	27713567.93	5.55	1 (ref.)	
	Incident MASLD	18,662 / 166,104	1153773.24	16.17	3.30	3.25–3.35
	Regressed MASLD	15,920 / 139,980	970866.91	16.40	2.87	2.82–2.92
	Persistent MASLD	41,070 / 243,685	1646900.1	24.94	5.28	5.22–5.34
Men	Never MASLD	65,524 / 1,818,089	13056351.47	5.02	1 (ref.)	
	Incident MASLD	12,161 / 132,599	929826.82	13.08	3.01	2.95–3.07
	Regressed MASLD	10,566 / 110,281	770893.93	13.71	2.70	2.65–2.76
	Persistent MASLD	32,900 / 211,864	1441783.35	22.82	5.26	5.19–5.33
Women	Never MASLD	88,299 / 2,029,950	14657216.46	6.02	1 (ref.)	
	Incident MASLD	6501 / 33,505	223946.42	29.03	3.90	3.80-4.00
	Regressed MASLD	5354 / 29,699	199972.98	26.77	3.19	3.10–3.28
	Persistent MASLD	8170 / 31,821	205116.74	39.83	5.09	4.97–5.21
Aged 20–39 years	Never MASLD	10,007 / 1,092,040	7991790.21	1.25	1 (ref.)	
	Incident MASLD	3110 / 56,399	403997.06	7.70	5.89	5.66–6.13
	Regressed MASLD	1643 / 32,115	230643.89	7.12	5.21	4.95–5.49
	Persistent MASLD	9646 / 80,845	563508.45	17.12	12.60	12.25–12.96
Aged 40–64 years	Never MASLD	98,391 / 2,270,062	16400926.95	6.00	1 (ref.)	
	Incident MASLD	12,221 / 93,148	642978.15	19.01	3.37	3.31–3.44
	Regressed MASLD	10,758 / 88,310	613203.28	17.54	2.98	2.92–3.04
	Persistent MASLD	26,461 / 142,341	954993.16	27.71	5.09	5.02–5.17
Aged ≥ 65 years	Never MASLD	45,425 / 485,937	3320850.77	13.68	1 (ref.)	
	Incident MASLD	3331 / 16,557	106798.03	31.19	2.42	2.34–2.51
	Regressed MASLD	3519 / 19,555	127019.74	27.70	2.12	2.05–2.20
	Persistent MASLD	4963 / 20,499	128398.48	38.65	3.07	2.98–3.16

*T2DM* type 2 diabetes mellitus,* MASLD* metabolic dysfunction associated steatotic liver disease,* IR* incident rate,* PYS* person-years,* HR* hazard ratio,* CI* confidence interval

*Adjusted for age, sex, alcohol drink, smoking, and regular exercise

### Incident CVD in individuals with T2DM according to the changes in MASLD

A total of 636,520 individuals with T2DM but without preexisting CVD were included in the analysis (Fig. 1B). During a median follow-up period of 6.2 years, 64,702 individuals (10.2%) developed CVD. Compared to those without incident CVD, individuals who experienced CVD events were significantly older and had a higher waist circumference, elevated liver enzymes, and impaired kidney function. Their mean body mass index (BMI) was higher (all *P* < 0.001, Supplementary Table 2^2^. However, there was no significant difference in the proportion of current smokers or the prevalence of dyslipidemia between individuals with and without incident CVD. When categorized changes in FLI, the proportions of individuals classified as never MASLD, incident MASLD, regressed MASLD, and persistent MASLD was 69.6%, 5.5%, 9.1%, and 15.8%, respectively (Table 2). Multivariate Cox analysis showed an increased CVD (composite of MI, stroke, and HF) risk across all MASLD groups compared to the never MASLD group. The adjusted HR were 1.12 (95% CI = 1.09–1.15) for regressed MASLD, and 1.18 (95% CI = 1.14–1.22) for incident MASLD, and the highest risk was observed in individuals with persistent MASLD (HR = 1.23, 95% CI = 1.20–1.26). Subgroup analyses demonstrated similar trends across the different CVD outcomes. Individuals with persistent MASLD had the highest risk for MI (HR = 1.15, 95% CI = 1.10–1.20) and HF (HR = 1.28, 95% CI = 1.25–1.32). The risk of stroke was comparable between the persistent MASLD (HR = 1.14, 95% CI = 1.09–1.19) and incident MASLD (HR = 1.14, 95% CI = 1.07–1.21). When stratified by sex, similar associations were observed in both men and women (Supplementary Table 3). In age-stratified analyses, the risk of CVD was significantly increased in individuals with persistent MASLD across all age groups. However, elevated CVD risk associated with incident or regressed MASLD was primarily observed in middle-aged and older individuals.

**Table 2:** Risk of incident CVD by MASLD status change in T2DM populations

Outcomes	Groups	No. of incident disease/No. in group	Duration, years	IR (per 1000 PYS)	HR*	95% CI
MI	Never MASLD	11,734 / 442,941	2704673.6	4.34	1 (ref.)	
	Incident MASLD	931 / 35,122	211564.3	4.40	1.23	1.06–1.21
	Regressed MASLD	1434 / 58,116	350975.3	4.09	1.04	0.98–1.10
	Persistent MASLD	2371 / 100,341	599254.1	3.96	1.15	1.10–1.20
Stroke	Never MASLD	14,581 / 442,941	2692524.1	5.42	1 (ref.)	
	Incident MASLD	1132 / 35,122	210661.8	5.37	1.14	1.07–1.21
	Regressed MASLD	1837 / 58,116	349082.5	5.26	1.11	1.06–1.17
	Persistent MASLD	2725 / 100,341	597396.2	4.56	1.14	1.09–1.19
Heart failure	Never MASLD	28,864 / 442,941	2678868.3	10.77	1 (ref.)	
	Incident MASLD	2296 / 35,122	209486.2	10.96	1.21	1.16–1.26
	Regressed MASLD	3570 / 58,116	347709.6	10.27	1.13	1.09–1.17
	Persistent MASLD	5747 / 100,341	594093.9	9.67	1.28	1.25–1.32
Composite outcome of MI, stroke, and heart failure	Never MASLD	46,091 / 442,941	2626840.6	17.55	1 (ref.)	
	Incident MASLD	3646 / 35,122	205290.7	17.76	1.18	1.14–1.22
	Regressed MASLD	5778 / 58,116	340856.9	16.95	1.12	1.09–1.15
	Persistent MASLD	9187 / 100,341	583497.8	15.74	1.23	1.20–1.26

*T2DM* type 2 diabetes mellitus,* MASLD* metabolic dysfunction associated steatotic liver disease,* IR* incident rate,* PYS* person-years,* HR* hazard ratio,* CI* confidence interval,* MI* myocardial infarction

*Adjusted for age, sex, alcohol drink, smoking, and regular exercise

### Overall mortality in individuals with T2DM according to the changes in MASLD

Among 822,673 individuals with T2DM, 66,503 (8.08%) died during the follow-up period. As shown in Table 3, overall mortality was significantly higher in individuals with incident, regressed, and persistent MASLD compared to those without MASLD. The HRs for mortality were 1.04 (95% CI = 1.01–1.07) for incident MASLD, 1.06 (95% CI = 1.03–1.09) for regressed MASLD, and 1.13 (95% CI = 1.08–1.14) for persistent MASLD. This trend was consistent in women, whereas in men, only persistent MASLD was associated with an increased risk of mortality. When stratified by age, both incident and persistent MASLD were associated with increased overall mortality in middle-aged and older individuals. However, in younger individuals with T2DM, neither incident nor persistent MASLD showed a significant increase in mortality risk. Notably, individuals with regressed MASLD in the young age group exhibited a significantly lower risk of mortality (HR = 0.34, 95% CI = 0.17–0.68).

**Table 3:** Risk of overall mortality by MASLD status change in T2DM populations

	Groups	No. of incident disease/No. in group	Duration, years	IR (per 1000 PYS)	HR*	95% CI
Overall	Never MASLD	49,726 / 577,762	3524381.0	14.11	1 (ref.)	
	Incident MASLD	3354 / 45,677	275401.4	12.18	1.04	1.01–1.07
	Regressed MASLD	5689 / 74,632	451601.3	12.60	1.06	1.03–1.09
	Persistent MASLD	7734 / 124,602	746821.4	10.36	1.11	1.08–1.14
Men	Never MASLD	29,646 / 277,451	1672125.9	17.73	1(ref.)	
	Incident MASLD	2175 / 28,037	168395.6	12.92	1.01	0.96–1.05
	Regressed MASLD	3772 / 47,971	288463.6	13.08	1.02	0.99–1.06
	Persistent MASLD	5453 / 88,607	529669.5	10.30	1.08	1.05–1.11
Women	Never MASLD	20,080 / 300,311	1852255.1	10.84	1 (ref.)	
	Incident MASLD	1179 / 17,640	107005.9	11.02	1.09	1.03–1.16
	Regressed MASLD	1917 / 26,661	163137.7	11.75	1.13	1.08–1.19
	Persistent MASLD	2281 / 35,995	217151.9	10.50	1.18	1.13–1.23
Aged 20–39 years	Never MASLD	85 / 9485	58596.2	1.45	1 (ref.)	
	Incident MASLD	11 / 1556	9479.4	1.16	0.72	0.38–1.35
	Regressed MASLD	9 / 2599	15979.3	0.56	0.34	0.17–0.68
	Persistent MASLD	89 / 9819	58488.4	1.52	0.93	0.69–1.25
Aged 40–64 years	Never MASLD	9771 / 316,116	1961494.6	4.98	1 (ref.)	
	Incident MASLD	940 / 28,802	175372.8	5.36	1.10	1.03–1.17
	Regressed MASLD	1557 / 47,307	288793.5	5.39	1.08	1.02–1.14
	Persistent MASLD	2728 / 84,332	508406.7	5.37	1.17	1.12–1.22
Aged ≥ 65 years	Never MASLD	39,870 / 252,161	1504290.2	26.50	1 (ref.)	
	Incident MASLD	2403 / 15,319	90549.2	26.54	1.02	0.98–1.06
	Regressed MASLD	4123 / 24,726	146828.5	28.08	1.06	1.02–1.09
	Persistent MASLD	4917 / 30,451	179926.3	27.33	1.08	1.05–1.11

*T2DM* Type 2 diabetes mellitus,* MASLD* metabolic dysfunction associated liver disease,* IR* incident rate,* PYS* person-years,* HR* hazard ratio,* CI* confidence interval

*Adjusted for age, sex, alcohol drink, smoking, and regular exercise

## Discussion

This nationwide cohort study demonstrated that individuals with FLI-based estimation of MASLD had an increased risk of developing T2DM, with the risk progressively increasing from regressed MASLD, incident MASLD compared to those who never had MASLD. Individuals with persistent MASLD as assessed by FLI exhibited a 5.28-fold higher risk of T2DM, with the greatest risk observed in the younger population (age 20–39 years). Among individuals with preexisting T2DM, persistent MASLD was associated with an increased the risk of incident CVD, including MI, stroke, and HF. The heighted CVD risk remained consistent across all age groups. Moreover, even individuals with incident or regressed MASLD assessed by FLI exhibited an elevated risk of CVD. Both persistent MASLD and temporal MASLD including both incident and regressed cases, were associated with increased overall mortality in the middle-aged and older individuals.

The current study has several clinical implications. First, this study provides epidemiological evidence supporting an association between MASLD and type 2 diabetes in the general population, based on a nationwide cohort. Given the close association between T2DM and MASLD, and the central role of insulin resistance as a shared pathogenic mechanism in both conditions [29], it is expected that individuals with MASLD are at an elevated risk of developing T2DM. Meta-analyses have demonstrated that MASLD is associated with an approximately two-fold increased risk of developing T2DM, independent of obesity and other common metabolic risk factors [30, 31]. Similarly, individuals with MASLD had a 3.83-fold increased risk of developing T2DM (95% CI = 3.81–3.86). With a longer duration of follow-up (median 7.3 years) and a substantially larger cohort (*n* = 4,397,808) compared to previous studies (median 5 years, *n* = 501,022) [30, 31], our study provides robust evidence supporting the increased risk for incident T2DM in a population with MASLD assessed by FLI.

Second, we examined the impact of dynamic changes in FLI-based estimation of MASLD on incident T2DM. The risk increased progressively from regressed MASLD to incident MASLD, with the highest risk observed in persistent MASLD. These findings underscore the critical role of persistent MASLD as a key determinant of T2DM risk and highlight the substantial impact of temporal changes in MASLD on T2DM development. Several observational cohort studies have reported that temporal changes in MASLD status are associated with varying risks of incident T2DM. Depending on the cohort population and covariates considered, persistent MASLD has been shown to increase the risk for incident T2DM by 1.50- to 7.38-fold, independent of body weight over time [32–35]. Most importantly, our study highlights that the risk of incident T2DM is disproportionately higher in younger individuals (aged 20–39 years) with persistent MASLD. Previous longitudinal studies have demonstrated the rapid progression of MASLD in adolescents and young adults [36, 37], suggesting a potentially more aggressive phenotype in these populations. Given that metabolic abnormalities, such as disturbances in branched chain and aromatic amino acid metabolism, can precede the development of MASLD by at least ten years [38], persistent MASLD in younger individuals may reflect a longer cumulative exposure to metabolic dysfunction and mitochondrial dysregulation. Our finding aligns with the observed rapid increase in MASLD prevalence among younger population with T2DM [3], emphasizing the importance of early identification and intervention in this high-risk group. Although we did not analyze longitudinal changes in body weight or waist circumference, which might have provided further mechanistic insights, previous results suggest that modifications in MASLD status may influence diabetes risk through liver-specific mechanisms [34]. Conversely, previous studies have reported that regressed or improved MASLD status was associated with either a significant reduction [35], or no significant change in incident T2DM risk, which contrasts with our finding [33, 34]. In the current study, we defined regressed MASLD based on repeated laboratory tests conducted at a 2-year interval. This suggests that even individuals with temporarily regressed MASLD may remain at risk for incident T2DM, highlighting the need for continued monitoring and follow-up for T2DM development.

Third, our study demonstrated that individuals with T2DM and persistent MASLD had a higher risk of CVD compared to those who never had MASLD, further expanding our understanding of the link between MASLD and CVD. MASLD exacerbates insulin resistance in both liver and peripheral tissues, contributes to atherogenic dyslipidemia, and triggers systemic and hepatic inflammation, all of which may collectively drive the development of CVD [12, 39]. Despite growing evidence linking MASLD to chronic vascular complications, the causal relationship between changes in MASLD status and CVD risk has not been clearly established. To our knowledge, this is the first study to examine the association between dynamic changes in MASLD and the risk of incident CVD among individuals with type 2 diabetes in a large-scale population. Utilizing nationwide cohort data and implementing a 1-year lag period to exclude individuals with preexisting CVD, we found that the association between persistent MASLD and CVD risk remained robust across different CVD events—including MI, ischemic stroke, and HF—and as well as across various subgroups. These findings highlight the clinical significance of persistent MASLD in identifying individuals with T2DM who are at an elevated risk for CVD. Similarly to their impact on the development T2DM, both incident and regressed MASLD were associated with an increased risk of incident CVD events. This highlights the necessity of surveillance and proactive CVD risk management in these populations.

Fourth, the results of the current study elucidate the impact of dynamic changes in MASLD status on overall mortality in individuals with established T2DM. Notably, both persistent and incident MASLD were associated with increased overall mortality, particularly in middle-aged and older individuals. While the specific causes of death could not be analyzed due to the privacy restrictions, prior studies have consistently identified CVD as the leading cause of mortality in patients with MASLD [40–42], which align with our findings. These results imply the necessity of a more proactive and systematic approach to evaluating T2DM-related comorbidities and mortality in patients with MASLD. Early identification and timely, aggressive intervention may be crucial in mitigating adverse outcomes in this high-risk population.

Despite the clinical significance of our study, we acknowledged several limitations. First, although FLI is a well-validated prediction model for steatotic liver disease and an appropriate cutoff was applied, it does not replace histological confirmation, and the severity of MASLD was not assessed. Although the FLI is a validated and widely used tool in large-scale epidemiological studies, it remains an indirect marker and may not fully capture true changes in liver fat content, particularly when its components are influenced by lifestyle or pharmacological interventions. Therefore, the potential for misclassification of hepatic steatosis status cannot be excluded. Moreover, BMI and waist circumference, which are important predictors of T2DM, CVD, and mortality, were not included as covariates in our models to avoid multicollinearity with the FLI. Thus, we cannot completely exclude the possibility that the observed associations were partially driven by adiposity measures rather than MASLD itself. Second, we were unable to account for changes in T2DM management modalities, which could influence the risk of comorbidities. Due to the limitations of the available data, diabetes was defined based on ICD-10 diagnostic codes and fasting plasma glucose levels, without systematic screening using glycated hemoglobin or oral glucose tolerance test. Therefore, the possibility of underdiagnosis or misclassification of diabetes status cannot be excluded. The unexpectedly higher prevalence of FLI assessed MASLD in individuals without diagnosed T2DM may reflect the broader and more heterogeneous nature of this group, which likely included individuals with undetected metabolic risks or prediabetes. Higher MASLD regression in individuals with T2DM may be partly attributed to the use of glucose-lowering agents such as sodium-glucose cotransporter-2 inhibitors or glucagon-like peptide-1 receptor agonists, which can improve hepatic steatosis independently of lifestyle changes. Third, due to the lack of available data, we could not assess the potential contributions of gut dysbiosis, genetic predisposition, or lifestyle factors beyond smoking and alcohol consumption. The Cox regression models adjusted only for demographic and lifestyle factors, and the lack of adjustment for clinical variables may have influenced the associations with CVD and mortality outcomes. Moreover, prior CVD was not included as a covariate in the mortality analysis, which may have confounded the observed associations. As this study is observational in nature, no causal relationship can be established between dynamic changes in MASLD and incident CVD. Fifth, survivorship bias may exist, as individuals who did not survive until the re-examination period may not have been included in the analysis. Sixth, since our study was conducted in a Korean population, the findings may not be generalizable to other ethnic or regional populations. Additionally, MASLD status was assessed only during health examinations conducted in 2009 and 2011, and changes in MASLD status thereafter were not captured. Therefore, our results reflect associations based on MASLD status during this specific period, and may not fully represent the incidence, persistence, or regression of MASLD during the entire follow-up. Cause-specific mortality data were not available in our dataset. As a result, we were unable to distinguish whether the associations observed were primarily driven by cardiovascular, cancer-related, or other causes of death. Lastly, the study was conducted under the conditions and data access policies in place at the time of project approval. While current NHIS regulations do not permit further access beyond the original research period, the analytic approach, cohort definitions, and statistical methods are fully documented and reproducible in principle using similar data.

## Conclusions

This nationwide cohort study demonstrated that both persistent and temporal MASLD as assessed by FLI significantly increase the risk of new-onset T2DM in the general population and further elevate the risks of CVD and overall mortality among individuals with T2DM. These findings highlight the necessity for physicians to assess MASLD status systematically and emphasize the importance of continuous screening strategies to identify and manage comorbidities, particularly in individuals with T2DM.

## Electronic supplementary material

Below is the link to the electronic supplementary material.

Supplementary Material 1.

## Data Availability

The dataset analyzed during the current study was obtained from the Korean National Health Insurance Service (NHIS) under project code NHIS-2023-1-786. Due to NHIS data access regulations, the authors no longer have access to the dataset, and data cannot be shared. Interested researchers may apply for access to similar data through the NHIS data request system (https://nhiss.nhis.or.kr), subject to approval and current policy limitations.

## Abbreviations

MASLD: Metabolic dysfunction-associated steatotic liver disease
HCC: Hepatocellular carcinoma
CVD: Cardiovascular disease
MI: Myocardial infarction
HF: Heart failure
T2DM: Type 2 diabetes mellitus
NHIS: National health insurance service
FLI: Fatty liver index
ICD-10: International statistical classification of diseases and related health problems, 10th revision
HR: Hazard ratio
95% CI: 95% confidence interval
