# Supplementary material for: Association of temporal MASLD with type 2 diabetes, cardiovascular disease and mortality
Source: Cardiovasc Diabetol. 2025 Jul 15;24:289. doi: 10.1186/s12933-025-02824-3 (PMC12261669; doi:10.1186/s12933-025-02824-3)
Supplement: Supplementary file 1 — Supplementary Material 1. [file 12933_2025_2824_MOESM1_ESM.docx]

Supplementary Figure 1. Study design summarizing participant selection and follow-up

Health checkups conducted from January 1, 2009, to December 31, 2012, were screened for patients with type 2 diabetes. Cases with CVD or death occurring within one year before or after repeated health checkups were excluded (gray box). Outcomes, including CVD and mortality, were tracked until December 31, 2010 (black arrow).

Supplementary Table 1. Baseline characteristics of the study population by incident T2DM

|  | Overall  (n=4,397,808) | Individuals without incident T2DM  (n=4,168,333) | Individuals with incident T2DM  (n=229,475) | P value |
| --- | --- | --- | --- | --- |
| Sex, men, n (%) | 2,272,833 (51.7) | 2,151,682 (51.6) | 121,151 (n=52.8) | <.0001 |
| Age, y | 48.0 ± 13.3 | 47.6 ± 13.2 | 55.6 ± 12.3 | <.0001 |
| Low income, n (%) | 760,261 (17.3) | 714,467 (17.1) | 45,794 (20.0) | <.0001 |
| Current smoking, n (%) | 935,155 (21.3) | 882,878 (21.2) | 52,277 (22.8) | <.0001 |
| Light alcohol consumption, n (%) | 1,952,415 (44.4) | 1,867,732 (44.8) | 84,683 (36.9) | <.0001 |
| Regular exercise, n (%) | 849,221 (19.3) | 802,932 (19.3) | 46,289 (20.2) | <.0001 |
| Obesity, n (%) | 1,337,201 (30.4) | 1,208,806 (29.0) | 128,395 (56.0) | <.0001 |
| Central obesity, n (%) | 746,297 (17.0) | 656,816 (15.8) | 89,481 (39.0) | <.0001 |
| Hypertension, n (%) | 1,017,557 (23.1) | 910,047 (21.8) | 107,510 (46.9) | <.0001 |
| Dyslipidemia, n (%) | 750,741 (17.1) | 673,869 (16.2) | 76,872 (33.5) | <.0001 |
| BMI, kg/m^2^ | 23.6 ± 3.1 | 23.4 ± 3.1 | 25.7 ± 3.4 | <.0001 |
| WC, cm | 79.5 ± 8.8 | 79.2 ± 8.7 | 85.3 ± 8.6 | <.0001 |
| Fasting glucose, mg/dL | 92.4 ± 10.8 | 91.9 ± 10.5 | 101.9 ± 12.3 | <.0001 |
| Systolic blood pressure, mmHg | 121.3 ± 14.4 | 121.0 ± 14.3 | 127.3 ± 14.8 | <.0001 |
| Diastolic blood pressure, mmHg | 75.7 ± 9.7 | 75.5 ± 9.7 | 78.8 ± 9.9 | <.0001 |
| Total cholesterol, mg/dL | 195.7 ± 35.4 | 195.2 ± 35.2 | 205.1 ± 38.5 | <.0001 |
| HDL cholesterol, mg/dL | 55.5 ± 17.5 | 55.8 ± 17.6 | 51.2 ± 15.7 | <.0001 |
| LDL cholesterol, mg/dL | 115.7 ± 32.7 | 115.4 ± 32.5 | 121.9 ± 36.1 | <.0001 |
| Triglyceride, mg/dL | 106.4 (106.3-106.5) | 104.7 (104.6-104.8) | 141.6 (41.3-141.9) | <.0001 |
| AST, IU/ml | 22.6 (22.5-22.6) | 22.4 (22.3-22.4) | 25.7 (25.7-25.8) | <.0001 |
| ALT, IU/ml | 19.9 (19.9-20.0) | 19.6 (19.5-19.6) | 26.2 (26.1-26.3) | <.0001 |
| γGT, IU/ml | 23.4 (23.3-23.4) | 22.9 (22.9-23.0) | 32.5 (32.-32.6) | <.0001 |
| eGFR, mL/min/1.73 m^2^ | 89.6 ± 37.6 | 89.8 ± 37.7 | 86.7 ± 36.0 | <.0001 |
| Follow-up duration, years | 7.3 (7.1-7.6) | 7.4 (7.2-7.6) | 4.5 (2.6-6.0) | <.0001 |

T2DM, type 2 diabetes mellitus; BMI, body mass index; WC, waist circumference; HDL cholesterol, high density lipoprotein cholesterol; LDL cholesterol, low density lipoprotein cholesterol; AST, aspartate aminotransferase; ALT, alanine transaminase; γGT, gamma glutamyl peptidase; eGFR, estimated glomerular filtration rate

Supplementary Table 2. Baseline characteristics of the study population by incident cardiovascular disease **(composite of myocardial infarction, stroke, and heart failure)**

|  | Overall  (n=636,520) | Individuals without incident CVD (n=571,818) | Individuals with incident CVD  (n=64,702) | P value |
| --- | --- | --- | --- | --- |
| Sex, men, n (%) | 352,628 (55.4) | 317,143 (55.5) | 35,485 (54.8) | 0.0027 |
| Age, years | 59.3 ± 10.9 | 58.6 ± 10.8 | 65.3 ± 10.0 | <.0001 |
| Low income, n (%) | 128,242 (20.2) | 114,364 (20.0) | 13,878 (21.5) | <.0001 |
| Current smoking, n (%) | 127,704 (20.1) | 114,758 (20.1) | 12,946 (20.0) | 0.7165 |
| Light alcohol consumption, n (%) | 227,335 (35.7) | 209,372 (36.6) | 17,963 (27.8) | <.0001 |
| Regular exercise, n (%) | 152,256 (23.9) | 138,580 (24.2) | 13,676 (21.1) | <.0001 |
| Obesity, n (%) | 299,577 (47.1) | 270,705 (47.3) | 28,872 (44.6) | <.0001 |
| Central obesity, n (%) | 231,978 (36.4) | 206,015 (36.0) | 25,963 (40.1) | <.0001 |
| Hypertension, n (%) | 373,978 (58.8) | 328,471 (57.4) | 45,507 (70.3) | <.0001 |
| Dyslipidemia, n (%) | 299,652 (47.1) | 269,301 (47.1) | 30,351 (46.9) | 0.3672 |
| T2DM duration ≥5 years, n (%) | 309998(48.7) | 269,741 (47.2) | 40,257 (62.2) | <.0001 |
| BMI, kg/m^2^ | 25.0 ± 3.3 | 25.0 ± 3.3 | 24.8 ± 3.3 | <.0001 |
| WC, cm | 85.0 ± 8.5 | 84.9 ± 8.5 | 85.6 ± 8.5 | <.0001 |
| Fasting glucose, mg/dL | 143.6 ± 47.3 | 143.5 ± 46.6 | 144.5 ± 53.0 | <.0001 |
| Systolic blood pressure, mmHg | 127.7 ± 15.0 | 127.5 ± 14.9 | 130.1 ± 16.0 | <.0001 |
| Diastolic blood pressure, mmHg | 77.8 ± 9.8 | 77.8 ± 9.7 | 77.8 ± 10.1 | 0.7867 |
| Total cholesterol, mg/dL | 188.7 ± 40.3 | 188.7 ± 40.2 | 188.9 ± 41.5 | 0.201 |
| HDL cholesterol, mg/dL | 50.4 ± 14.6 | 50.4 ± 14.5 | 49.6 ± 15.8 | <.0001 |
| LDL cholesterol, mg/dL | 107.8 ± 36.7 | 107.7 ± 36.7 | 108.5 ± 37.2 | <.0001 |
| Triglyceride, mg/dL | 134.8 (134.6-135.0) | 134.6 (134.4-134.8) | 136.9 (136.3-137.4) | <.0001 |
| AST, IU/ml | 24.4 (24.3-24.4) | 24.5 (24.4-24.5) | 23.8 (23.7-23.9) | <.0001 |
| ALT, IU/ml | 24.6 (24.5-24.6) | 24.8 (24.7-24.8) | 22.6 (22.4-22.7) | <.0001 |
| γGT, IU/ml | 30.3 (30.3-30.4) | 30.4 (30.4-30.5) | 28.9 (28.8-29.2) | <.0001 |
| eGFR, mL/min/1.73 m^2^ | 87.0 ± 39.3 | 87.7 ± 39.4 | 81.0 ± 38.5 | <.0001 |
| Follow-up duration, years | 6.2 (5.0-7.1) | 6.4 (5.2-7.2) | 3.9 (2.3-5.3) | <.0001 |

CVD, cardiovascular disease; T2DM, type 2 diabetes mellitus; BMI, body mass index; WC, waist circumference; HDL cholesterol, high density lipoprotein cholesterol; LDL cholesterol, low density lipoprotein cholesterol; AST, aspartate aminotransferase; ALT, alanine transaminase; γGT, gamma glutamyl peptidase; eGFR, estimated glomerular filtration rate

Supplementary Table 3. Subgroup analysis of incident CVD **(composite of MI, stroke, and heart failure)** risk by MASLD status change in the T2DM population

|  |  | Groups | No. of incident disease  /No. in group | Duration, year | IR (per  1000  PYS) | HR* | 95% CI |
| --- | --- | --- | --- | --- | --- | --- | --- |
| MI | Overall | Never MASLD | 11,734 / 442,941 | 2704673.6 | 4.34 | 1 (Ref.) |  |
|  |  | Incident MASLD | 931 / 35,122 | 211564.3 | 4.40 | 1.13 | 1.06-1.21 |
|  |  | Regressed MASLD | 1,434 / 58,116 | 350975.3 | 4.09 | 1.04 | 0.98-1.10 |
|  |  | Persistent MASLD | 2,371 / 100,341 | 599254.1 | 3.96 | 1.15 | 1.10-1.20 |
|  | Men | Never MASLD | 6,155 / 216,953 | 1312463.9 | 4.69 | 1 (Ref.) |  |
|  |  | Incident MASLD | 611 / 22,417 | 134549.9 | 4.54 | 1.16 | 1.07-1.26 |
|  |  | Regressed MASLD | 952 / 38,922 | 233951.7 | 4.07 | 1.03 | 0.96-1.10 |
|  |  | Persistent MASLD | 1,751 / 74,336 | 442847.6 | 3.95 | 1.16 | 1.10-1.23 |
|  | Women | Never MASLD | 5,579 / 225,988 | 1392209.7 | 4.01 | 1 (Ref.) |  |
|  |  | Incident MASLD | 320 / 12,705 | 77014.4 | 4.16 | 1.09 | 0.97-1.22 |
|  |  | Regressed MASLD | 482 / 19,194 | 117023.7 | 4.12 | 1.06 | 0.97-1.17 |
|  |  | Persistent MASLD | 620 / 26,005 | 156406.4 | 3.96 | 1.11 | 1.02-1.21 |
|  | Aged 20-39 years | Never MASLD | 62 / 9,253 | 57021.3 | 1.09 | 1 (Ref.) |  |
|  |  | Incident MASLD | 6 / 1,516 | 9230.3 | 0.65 | 0.56 | 0.24-1.29 |
|  |  | Regressed MASLD | 19 / 2,538 | 15564.2 | 1.22 | 1.04 | 0.62-1.74 |
|  |  | Persistent MASLD | 113 / 9,543 | 56606.4 | 2.00 | 1.70 | 1.25-2.32 |
|  | Aged 40-64 years | Never MASLD | 5,053 / 269,317 | 1663051.9 | 3.04 | 1 (Ref.) |  |
|  |  | Incident MASLD | 510 / 24,176 | 146304.6 | 3.49 | 1.18 | 1.07-1.29 |
|  |  | Regressed MASLD | 794 / 40,235 | 244132.0 | 3.25 | 1.07 | 1,10-1.16 |
|  |  | Persistent MASLD | 1,488 / 71,883 | 430458.2 | 3.46 | 1.20 | 1.13-1.27 |
|  | Aged ≥65 years | Never MASLD | 6,619 / 164,371 | 984600.4 | 6.72 | 1 (Ref.) |  |
|  |  | Incident MASLD | 415 / 9,430 | 56029.4 | 7.41 | 1.11 | 1.01-1.23 |
|  |  | Regressed MASLD | 621 / 15,343 | 91279.2 | 6.80 | 1.01 | 0.93-1.10 |
|  |  | Persistent MASLD | 770 / 18,915 | 112189.5 | 6.86 | 1.04 | 0.96-1.12 |
| Stroke | Overall | Never MASLD | 14,581 / 442,941 | 2692524.1 | 5.42 | 1 (Ref.) |  |
|  |  | Incident MASLD | 1,132 / 35,122 | 210661.8 | 5.37 | 1.14 | 1.09-1.21 |
|  |  | Regressed MASLD | 1,837 / 58,116 | 349082.5 | 5.26 | 1.11 | 1.06-1.17 |
|  |  | Persistent MASLD | 2,725 / 100,341 | 597396.2 | 4.56 | 1.14 | 1.09-1.19 |
|  | Men | Never MASLD | 7,800 / 216,953 | 1305896.7 | 5.97 | 1 (Ref.) |  |
|  |  | Incident MASLD | 696 / 22,417 | 134144.6 | 5.19 | 1.08 | 1.00-1.17 |
|  |  | Regressed MASLD | 1,205 / 38,922 | 232785.7 | 5.18 | 1.08 | 1.02-1.15 |
|  |  | Persistent MASLD | 1,934 / 74,336 | 441881.2 | 4.38 | 1.10 | 1.04-1.16 |
|  | Women | Never MASLD | 6,781 / 225,988 | 1386627.4 | 4.89 | 1 (Ref.) |  |
|  |  | Incident MASLD | 436 / 12,705 | 76517.2 | 5.70 | 1.24 | 1.12-1.37 |
|  |  | Regressed MASLD | 632 / 19,194 | 116296.8 | 5.43 | 1.17 | 1.08-1.27 |
|  |  | Persistent MASLD | 791 / 26,005 | 155514.9 | 5.09 | 1.21 | 1.13-1.31 |
|  | Aged 20-39 years | Never MASLD | 41 / 9,253 | 57046.4 | 0.72 | 1 (Ref.) |  |
|  |  | Incident MASLD | 10 / 1,516 | 9219.7 | 1.08 | 1.38 | 0.69-2.75 |
|  |  | Regressed MASLD | 11 / 2,538 | 15575.6 | 0.71 | 0.89 | 0.46-1.74 |
|  |  | Persistent MASLD | 63 / 9,543 | 56712.3 | 1.11 | 1.38 | 0.93-2.05 |
|  | Aged 40-64 years | Never MASLD | 5,264 / 26,9317 | 1660595.6 | 3.17 | 1 (Ref.) |  |
|  |  | Incident MASLD | 503 / 24,176 | 146223.2 | 3.44 | 1.09 | 1.00-1.20 |
|  |  | Regressed MASLD | 858 / 40,235 | 243462.6 | 3.52 | 1.11 | 1.03-1.19 |
|  |  | Persistent MASLD | 1,466 / 71,883 | 429967.2 | 3.41 | 1.12 | 1.05-1.19 |
|  | Aged ≥65 years | Never MASLD | 9,276 / 164,371 | 974882.2 | 9.52 | 1 (Ref.) |  |
|  |  | Incident MASLD | 619 / 9,430 | 55218.8 | 11.21 | 1.18 | 1.09-1.28 |
|  |  | Regressed MASLD | 968 / 15,343 | 90044.3 | 10.75 | 1.12 | 1.05-1.20 |
|  |  | Persistent MASLD | 1,196 / 18,915 | 110716.7 | 10.80 | 1.25 | 1.08-1.22 |
| HF | Overall | Never MASLD | 28,864 / 442,941 | 2678868.3 | 10.77 | 1 (Ref.) |  |
|  |  | Incident MASLD | 2,296 / 35,122 | 209486.2 | 10.96 | 1.21 | 1.16-1.26 |
|  |  | Regressed MASLD | 3,570 / 58,116 | 347709.6 | 10.27 | 1.13 | 1.09-1.17 |
|  |  | Persistent MASLD | 5,747 / 100,341 | 594093.9 | 9.67 | 1.28 | 1.25-1.32 |
|  | Men | Never MASLD | 14,277 / 216,953 | 1301596.1 | 10.97 | 1 (Ref.) |  |
|  |  | Incident MASLD | 1,331 / 22,417 | 133653.5 | 9.96 | 1.14 | 1.08-1.21 |
|  |  | Regressed MASLD | 2,175 / 38,922 | 232244.4 | 9.37 | 1.07 | 1.03-1.12 |
|  |  | Persistent MASLD | 3,750 / 74,336 | 440268.4 | 8.52 | 1.19 | 1.15-1.23 |
|  | Women | Never MASLD | 14,587 / 225,988 | 1377272.2 | 10.59 | 1 (Ref.) |  |
|  |  | Incident MASLD | 965 / 12,705 | 75832.7 | 12.73 | 1.29 | 1.21-1.38 |
|  |  | Regressed MASLD | 1,395 / 19,194 | 115465.3 | 12.08 | 1.20 | 1.14-1.27 |
|  |  | Persistent MASLD | 1,997 / 26,005 | 153825.5 | 12.98 | 1.45 | 1.38-1.52 |
|  | Aged 20-39 years | Never MASLD | 174 / 9,253 | 56846.2 | 3.06 | 1 (Ref.) |  |
|  |  | Incident MASLD | 23 / 1,516 | 9201.6 | 2.50 | 0.78 | 0.51-1.21 |
|  |  | Regressed MASLD | 42 / 2,538 | 15527.8 | 2.70 | 0.83 | 0.59-1.17 |
|  |  | Persistent MASLD | 166 / 9,543 | 56548.0 | 2.94 | 0.93 | 0.75-1.15 |
|  | Aged 40-64 years | Never MASLD | 10,666 / 269,317 | 1655096.4 | 6.44 | 1 (Ref.) |  |
|  |  | Incident MASLD | 1,110 / 24,176 | 145489.8 | 7.63 | 1.27 | 1.19-1.35 |
|  |  | Regressed MASLD | 1,677 / 40,235 | 242796.2 | 6.91 | 1.13 | 1.08-1.19 |
|  |  | Persistent MASLD | 3,110 / 71,883 | 428218.3 | 7.26 | 1.29 | 1.24-1.35 |
|  | Aged ≥65 years | Never MASLD | 18,024 / 164,371 | 966925.7 | 18.64 | 1 (Ref.) |  |
|  |  | Incident MASLD | 1,163 / 9,430 | 54794.9 | 21.22 | 1.16 | 1.10-1.23 |
|  |  | Regressed MASLD | 1,851 / 15,343 | 89385.7 | 20.71 | 1.13 | 1.08-1.19 |
|  |  | Persistent MASLD | 2,471 / 18,915 | 109327.7 | 22.60 | 1.28 | 1.22-1.33 |
| **Composite outcome of MI, stroke, and heart failure** | Overall | Never MASLD | 46,091 / 442,941 | 2626840.6 | 17.55 | 1 (Ref.) |  |
|  |  | Incident MASLD | 3,646 / 35,122 | 205290.7 | 17.76 | 1.18 | 1.14-1.22 |
|  |  | Regressed MASLD | 5,778 / 58,116 | 340856.9 | 16.95 | 1.12 | 1.09-1.15 |
|  |  | Persistent MASLD | 9,187 / 100,341 | 583497.8 | 15.74 | 1.23 | 1.20-1.26 |
|  | Men | Never MASLD | 23,364 / 216,953 | 1274194.6 | 18.34 | 1 (Ref.) |  |
|  |  | Incident MASLD | 2,193 / 22,417 | 131005.0 | 16.74 | 1.13 | 1.08-1.18 |
|  |  | Regressed MASLD | 3,650 / 38,922 | 227780.6 | 16.02 | 1.08 | 1.04-1.12 |
|  |  | Persistent MASLD | 6,278 / 74,336 | 432588.3 | 14.51 | 1.17 | 1.14-1.20 |
|  | Women | Never MASLD | 22,727 / 225,988 | 1352645.9 | 16.80 | 1 (Ref.) |  |
|  |  | Incident MASLD | 1,453 / 12,705 | 74285.7 | 19.56 | 1.24 | 1.18-1.31 |
|  |  | Regressed MASLD | 2,128 / 19,194 | 113076.3 | 18.82 | 1.18 | 1.13-1.23 |
|  |  | Persistent MASLD | 2,909 / 26,005 | 150909.5 | 19.28 | 1.34 | 1.29-1.39 |
|  | Aged 20-39 years | Never MASLD | 249 / 9,253 | 56645.4 | 4.40 | 1 (Ref.) |  |
|  |  | Incident MASLD | 36 / 1,516 | 9165.6 | 3.93 | 0.84 | 0.59-1.20 |
|  |  | Regressed MASLD | 64 / 2,538 | 15468.8 | 4.14 | 0.88 | 0.66-1.15 |
|  |  | Persistent MASLD | 298 / 9,543 | 56210.3 | 5.30 | 1.13 | 0.96-1.34 |
|  | Aged 40-64 years | Never MASLD | 17,979 / 269,317 | 1632674.5 | 11.01 | 1 (Ref.) |  |
|  |  | Incident MASLD | 1,808 / 24,176 | 143419.7 | 12.61 | 1.20 | 1.14-1.26 |
|  |  | Regressed MASLD | 2,857 / 40,235 | 239137.3 | 11.95 | 1.12 | 1.08-1.16 |
|  |  | Persistent MASLD | 5,235 / 71,883 | 421571.9 | 12.42 | 1.24 | 1.20-1.28 |
|  | Aged ≥65 years | Never MASLD | 27,863 / 164,371 | 937520.7 | 29.72 | 1 (Ref.) |  |
|  |  | Incident MASLD | 1,802 / 9,430 | 52705.4 | 34.19 | 1.17 | 1.11-1.22 |
|  |  | Regressed MASLD | 2,857 / 15,343 | 86250.9 | 33.12 | 1.13 | 1.08-1.17 |
|  |  | Persistent MASLD | 3,654 / 18,915 | 105715.5 | 34.56 | 1.20 | 1.16-1.25 |

T2DM, type 2 diabetes mellitus; MASLD, metabolic dysfunction associated steatotic liver diasese; IR, incidence rate, PYS, person-years; HR, hazard ratio; CI, confidence interval; MI, myocardial infarction; HF, heart failure

*Adjusted for age, sex, alcohol drink, smoking, and regular exercise
